# Tocilizumab-Based Treatment of Microvascular Inflammation in Kidney Transplant Recipients: A Retrospective Study

**DOI:** 10.3389/ti.2025.14502

**Published:** 2025-05-16

**Authors:** Johan Noble, Giorgia Comai, Valeria Corredetti, Reda Laamech, Celine Dard, Thomas Jouve, Diane Giovannini, Audrey Le Gouellec, Shivani Wadnerkar, Paolo Cravedi, Della Apuzzo, Daniele Vetrano, Marco Busutti, Chiara Abenavoli, Paolo Malvezzi, Lionel PE Rostaing, Gaetano Lamanna

**Affiliations:** ^1^ Nephrology, Hemodialysis, Apheresis and Kidney Transplantation Department, University hospital Grenoble, Grenoble, France; ^2^ Univ. Grenoble Alpes, CNRS, Inserm, U 1209 CNRS UMR 5309, Team Epigenetis Immunity, Metabolism, Cell Signaling and Cancer, Institute for advanced Biosciences, Grenoble, France; ^3^ Precision Immunology Institute, Translational Transplant Research Center (TTRC), Icahn School of Medicine at Mount Sinai, New York, NY, United States; ^4^ Nephrology, Dialysis and Kidney Transplant Unit, IRCCS-Azienda Ospedaliero-Universitaria di Bologna, Bologna, Italy; ^5^ Department of Medical and Surgical Sciences (DIMEC), Alma Mater Studiorum-University of Bologna, Bologna, Italy; ^6^ Établissement Français du Sang Auvergne-Rhône-Alpes, HLA and immunogenetics Laboratory, Grenoble, France; ^7^ Anatomopathology Department, University hospital Grenoble, Grenoble, France; ^8^ Université Grenoble Alpes, CNRS, Grenoble INP, CHU Grenoble Alpes, TIMC-IMAG, Grenoble, France

**Keywords:** kidney transplantation, tocilizumab, microvascular inflammation, donor-specific antibody, chronic-active antibody-mediated rejection

## Abstract

Chronic-active antibody mediated rejection (caAMR) is the leading causes of long-term kidney graft failure. Tocilizumab (TCZ), an anti-IL-6 receptor antibody, has been suggested as a treatment, but data are conflicting. We retrospectively studied consecutive adult kidney transplant recipients with caAMR or microvascular inflammation (MVI) without Donor-Specific Antibodies (DSA) and without C4d deposition (MVI + DSA-C4d-), who received TCZ as first-line therapy in two European centers. Estimated glomerular filtration rate (eGFR) and DSA were assessed one-year before and after TCZ initiation. The study included 64 patients who received TCZ between July 2018 and September 2023. The eGFR trajectory significantly decreased after TCZ treatment (−1.2 ± 0.2 vs. 0.03 ± 0.2 mL/min/1.73 m^2^/month pre- vs. post-TCZ, respectively; p = 0.001). The percentage of patients with DSA decreased from 63.9% to 38.9% (p < 0.001), and the average MFI decreased from 9,537 to 7,250 (p = 0.001). In multivariate analysis, younger age (OR = 0.95, p = 0.02), MVI + DSA-C4d- phenotype (OR = 5.2, p = 0.01), and lower chronic glomerulopathy score (OR = 4.5, p = 0.02) were associated with TCZ response (trajectory ≥0 after TCZ). Patient survival was 98.4%, and graft survival was 93.7% at one-year. First-line TCZ therapy for caAMR or MVI + DSA-C4d- is associated with an improvement of eGFR trajectories, reduced DSA numbers and MFI and histological inflammation in glomeruli. These data suggest a potential benefit of TCZ in these settings.

## Graphical Abstract

**Figure:**
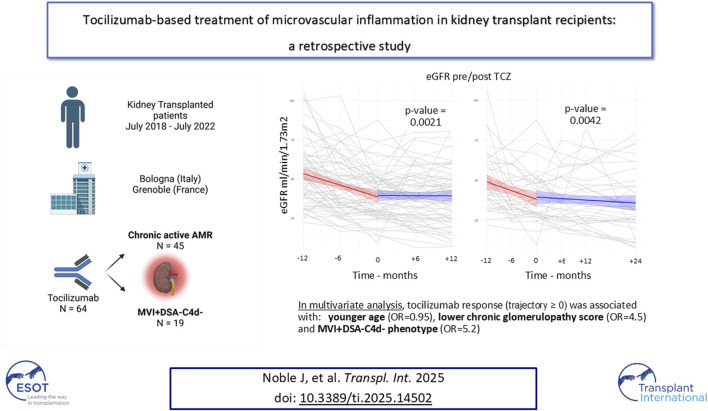

## Introduction

Chronic active Antibody-mediated rejection (caAMR) is a leading cause of immune-mediated allograft failure after kidney transplantation (KT) [1–3]. The key histological features of caAMR diagnosis demonstrate signs of a recent interaction between alloantibodies and vascular endothelium, i.e., microvascular inflammation (MVI) [glomerulitis(g) and peritubular capillaritis (ptc)], with chronic features such as transplant glomerulopathy (cg > 0) and/or severe peritubular capillary basement membrane multilayering [4, 5]. caAMR is primarily driven by the development of donor-specific antibodies (DSA), which can trigger complement activation and subsequent allograft injury due to direct endothelial cell damage and the recruitment of innate immune cells such as neutrophils, macrophages, and natural killer (NK) cells, which further exacerbate inflammation and tissue damage [6–8].

CaAMR appears to be a more complex and polymorphic entity, as some patients present with microvascular inflammation without DSA or C4d staining [9]. In 2022, the 16th Banff meeting for allograft pathology defined new phenotypes in patients presenting MVI: MVI-positive, DSA-negative and C4d-negative (MVI + DSA-C4d-) and probable AMR in patients DSA + but MVI below the threshold (i.e., g + ptc <2) [10]. In 2024, Sablik et al. showed that after reclassifications of patients according to the new Banff classification, those two newly defined phenotypes exhibit a worse graft survival at 5 years of the biopsy compared to the patients classified without AMR [9]. The physiopathology of MVI + DSA-C4d- lesions may be driven by NK cells and T-cells rather than antibodies [11–13].

Treatment of these conditions remains a major unmet need [14]. Despite recent evidence from a pilot safety phase II randomized clinical study showing efficacy of anti-CD38 depleting antibody on histological remission and renal function in 9 of 11 active and caAMR patients (defined according to 2019 Banff classification), there is no consensus on the optimal treatment [15].

Interleukin-6 (IL-6) is a pleiotropic cytokine implicated in promoting the activation and expansion of B and T cells, especially T-follicular helper cells, and in the initiation of the acute phase inflammatory response [16]. IL-6 also inhibits the induction of regulatory T cells and promotes their conversion into Th17 cells [16]. Blockade of the IL-6/IL-6 receptor axis, a well-established concept for the treatment of autoimmune diseases, is an attractive option to treat MVI progression through its effect on antibody secretion, T-cell regulatory/effector balance, and endothelial activation by DSA [17, 18].

Experimental data from a mouse model of skin transplantation have shown that IL-6 blockade decreased antibody rebound after a second skin graft, reduced pro-inflammatory cytokine, and increased regulatory T-cells [19].

Tocilizumab (TCZ, Actemra/RoActemra, Roche/Genentech, San Francisco, CA), an anti-IL-6 receptor humanized monoclonal antibody, has been proven to decrease immunodominant DSA (iDSA), C4d, microvascular inflammation and to stabilize transplant glomerulopathy in retrospective studies [20–22]. Those study included small retrospective, heterogeneous and monocentric cohorts. To date, there are no randomized studies assessing its efficacy in caAMR or MVI + DSA-C4d- [22–25]. The INTERCEPT study is a controlled open-label multi-center randomized clinical trial (RCT) in KT recipients to compare the efficacy of TCZ in caAMR. This study is still under recruitment [26].

A phase 2 randomized trial to assess the safety and efficacy of Clazakizumab (CLZ), an anti-IL-6 antibody in late antibody-mediated rejection showed a significant decrease of DSA MFI but failed to find a significant benefit in molecular and histologic scores of rejection [27]. Besides, CLZ was able to improve the estimated glomerular filtration rate (eGFR) trajectory, but it was associated with a high risk of gastrointestinal perforation. Therefore, the phase 3 IMAGINE trial tested a lower CLZ dose to reduce toxicity in accordance with the phase 2 trial gastrointestinal complications. However, it was discontinued early due to futility [28].

This is a retrospective multicentric study in two independent European transplant centers using TCZ as first-line therapy of KT patients with MVI including caAMR and MVI + DSA-C4d-.

## Materials and Methods

### Population Study

In this multicenter, retrospective study, we assessed all consecutive adult KT recipients between July 2018 and July 2022 from nephrology and KT departments of Grenoble (France) and Bologna (Italy). The study included all patients with histological signs of caAMR or MVI + DSA-C4d- according to the 2022 Banff classification and that received intravenous TCZ at a dose of 8 mg/kg monthly and/or subcutaneous at the dose of 162 mg/15 days. Patients presenting *de novo* or recurrence of glomerulopathy, acute tubular necrosis were excluded. In Grenoble, surveillance kidney biopsies were performed 1-year after starting TCZ.

Patients were monitored for renal function (eGFR calculated using the CKD-EPI formula), urine albumin‐to‐creatinine ratio (UACR) (g/g), adverse and severe adverse events from treatment, graft- and patient-survival rates, and DSA levels every 3 months. Patient were all followed at least 1 year following MVI treatment initiation and data were collected at last available follow-up.

TCZ was given off-label in the absence of validated therapeutic alternative. Regarding retrospective data assessment, all patients signed an informed consent form. The study was performed in accordance with the ethical standards of the 1964 Declaration of Helsinki and its later amendments. All medical data were collected from Grenoble database [CNIL (French National committee for data protection) approval number 1987785v0] and from Bologna KT Center informatics repository for clinical data (protocol number 244/2023/Sper/AOUBo).

### Immunosuppression Regimen

In both centers, all patients received induction with antithymocyte globulins or Basiliximab, methylprednisone (500 mg), and started peri-operatively with 1 g of mycophenolate mofetil or 720 mg mycophenolic acid.

Maintenance immunosuppression after KT consisted of tacrolimus or cyclosporine associated with mycophenolate mofetil/mycophenolic acid or everolimus. In Grenoble, steroids were rapidly tapered until withdrawal at month-3 except for highly sensitized patients (PRA more than 75%), patients who had experienced a previous acute rejection, patients with IgA nephropathy, or patients with circulating DSAs. In Bologna, steroids were rapidly tapered and maintained at prednisone 5 mg per day.

### Pathological Assessment of Graft Biopsy

MVI-positive biopsies, including caAMR and MVI + DSA-C4d- were diagnosed on biopsies performed for clinical indications, i.e., rising serum creatinine and/or *de novo* proteinuria and/or *de novo* DSA detection. C4d staining was performed for all biopsies. The kidney-graft sample was processed for light microscopy by fixing in alcohol-formol-acetic acid (AFA, fluid and embedded in paraffin). Specimens were stained with trichrome blue, hematoxylin eosin and safran, periodic acid-schiff reagent, C4d staining, and immunofluorescence studies, including direct immunofluorescence for immunoglobulins, immunoglobulin light chains, C3 and C1q fractions of the complement. The results of biopsies were all re-assessed according to the 2022 Banff classification of rejection [10].

### Anti-HLA Antibody Measurement

DSA were monitored at the time of each kidney biopsy, i.e., at MVI diagnosis and at 1-year post treatment. Screening of HLA Class I and II DSA in recipients was performed on sera using a Luminex platform with two different single antigen bead assay kits in the HLA department of Grenoble and of Bologna (Luminex Single Antigen assay, Immucor, Norcross, GA, United States and LABScreen, One Lambda Inc, Canoga Park, CA, United States) Screening and single-antigen assessment were performed every 6 months post-transplant and systematically at the time of a kidney biopsy. The retained mean fluorescence intensity (MFI) values corresponded to the manufacturers background corrected MFI value. Positivity for the Luminex analysis was defined as an MFI > 500.

### Interleukin-6 Measurement

IL-6 was assayed on frozen sera at the time of MVI diagnosis (before any treatments) and at 1-year post treatment initiation. In Grenoble, IL-6 quantitative dosage in sera was performed using a Lumipulse G600II system (Fujirebio, Tokyo, Japan).

### Statistical Analysis

The primary endpoint was the eGFR trajectory before and after TCZ initiation. To calculate and compare the trajectories of eGFR before and after TCZ, a mixed linear regression model with random intercepts was employed. The model included an interaction term between the time variable and the period variable, which allowed for the evaluation of differences in the trajectories between the “Before” and “After” periods. Five patients were diagnosed MVI-positive within the first-year post-transplantation. For the calculation of GFR trajectory, while most of studies remove patients from with end-stage renal disease from the eGFR trajectories, we assigned an eGFR value of 10 mL/min/1.73 m^2^for patients who experienced graft loss during follow-up. This value was chosen to reflect a realistic approximation of kidney function at the time of progression to end-stage kidney disease while maintaining the continuous nature of the variable. This approach avoids the introduction of extreme imputation values (e.g., 0 mL/min/1.73 m^2^ of eGFR), which could disproportionately skew the trajectory analysis, particularly for patients already in advanced stages of chronic kidney disease at baseline.

Secondary endpoints were the one-year death-censored graft survival rate, proteinuria (g/g of creatininuria) and DSA. Six patients were not classified because of a follow-up shorter than 1-year and were excluded of the analysis. Thirty-three patients did not reach 2-year of follow-up and were excluded for the 2-year eGFR trajectories analysis. Evolution of histological biopsies was analyzed only in the Grenoble-Alpes hospital where patients had follow-up biopsies at 1-year.

Continuous variables are presented as means ± standard deviations (SD) or as medians with quartiles [Q1–Q3] in cases of high dispersion. Qualitative data are reported as the numbers of patients/events and percentages. The Wilcoxon or the Kruskal--Wallis tests were used for continuous variables, the chi-squared test was used for categorical data. For paired categorical data we used the Stuart-Maxwell test. For graft survival analyses, a multivariate generalized logistic-regression model was run. A two-sided *p*-value of <0.05 was considered statistically significant. Statistical analyses were conducted using R statistical software [29].

## Results

### Baseline Characteristics

Between July 2018 and July 2024, 64 patients presented histological lesions of MVI on a for cause kidney biopsy performed after a median period of 58 [16–130] months post-transplantation. Patients’ age was 48.6 ± 14 years; female/male ratio was 0.4. Most of KT were deceased donor transplants (83.3%). The baseline characteristics of the cohort in both categories of MVI (i.e., caAMR and MVI + DSA-C4d-) are reported in Table 1. Chronic glomerulopathy lesions (cg score of ≥1) were present in 53.1% of patients, glomerulitis lesions (g score of ≥1) in 81.2%, and peritubular capilaritis (ptc score of ≥1) in 82.3% of patients. C4d staining was positive in 26.5% of patients. DSA were positive in 40 of patients (62.5%). eGFR at the time of biopsy was similar in caAMR and MVI + DSA-C4d- groups (41.1 ± 15 versus 37.9 ± 17, p = 0.378 respectively). We also compared the patients in both centers (Supplementary Table S1). Patients from Grenoble versus Bologna were significantly younger (44.8 versus 53.3 years, p = 0.02), received more often antithymoglobulin (100% versus 44.4%, p < 0.001), had less DSA at the time of biopsy (48.7% versus 84%, p = 0.01) and had earlier MVI diagnosis (median 35 months post-KT versus 154 months, p < 0.001).

**TABLE 1:** Baseline characteristics.

	Patient with TCZ as a first line therapy for MVI N = 64		
Variables	caAMR N = 45	MVI + DSA-C4d-N = 19	p-value
Age – years	48.8 ± 14	46.4 ± 14.7	0.562
Female Gender - N (%)	17 (37.8%)	9 (47.4%)	0.475
Pre-emptive transplantation – N (%)	2 (4.4%)	4 (21.1%)	0.037
Nephropathy– N (%) PKD Diabetes Vascular disease Autoimmune Unknown Other	10 (22.2%) 4 (8.9%) 3 (6.6%) 5 (11.1%) 15 (33.3%) 6 (13.3%)	5 (26.3%) 2 (10.6%) 1 (5.3%) 2 (10.6%) 7 (36.9%) 4 (21%)	0.778
Induction therapy Antithymoglobulin Basiliximab	35 (77.7%) 10 (22.2%)	17 (89.4%) 2 (10.5%)	0.325
Living donor – N (%)	8 (17.8%)	3 (15.8%)	0.847
Serum creatinine at the time of biopsy - µmol/L	177 ± 54.8	191 ± 130	0.234
eGFR at the time of biopsy – mL/min/1.73m^2^	41.1 ± 15	37.9 ± 17	0.378
Albuminuria at the time of biopsy – g/g of creatininuria	0.9 ± 1.1	1.4 ± 1.7	0.61
Time after transplant - months	34.8 [15–89]	28.2 [5–57]	0.217
Immunosuppression at the time of biopsy - Tacrolimus - Cyclosporine - MMF - Everolimus	32 (72.7%) 11 (25.0%) 38 (88.4%) 2 (4.7%)	18 (94.7%) 1 (5.3%) 14 (73.7%) 5 (26.3%)	0.067

AMR, anitbody mediated rejection; DSA, Donor-specific antibody; cg, chronic glomerulopathy; eGFR, estimated glomerular filtration rate; MVI, microvascular inflammation.

### Kidney Function

eGFR at the time of biopsy (before TCZ treatment) was 39.4 ± 16 mL/min/1.73 m^2^. The eGFR 1-year before TCZ treatment was 54.0 ± 19 mL/min/1.73 m^2^ and at 1 year after TCZ treatment was 40.8 ± 18 mL/min/1.73 m^2^. Among thirty-one patients (43%) with a follow-up of 2 years post-treatment, last eGFR was 43.6 ± 18 mL/min/1.73 m^2^.

**FIGURE 1:**
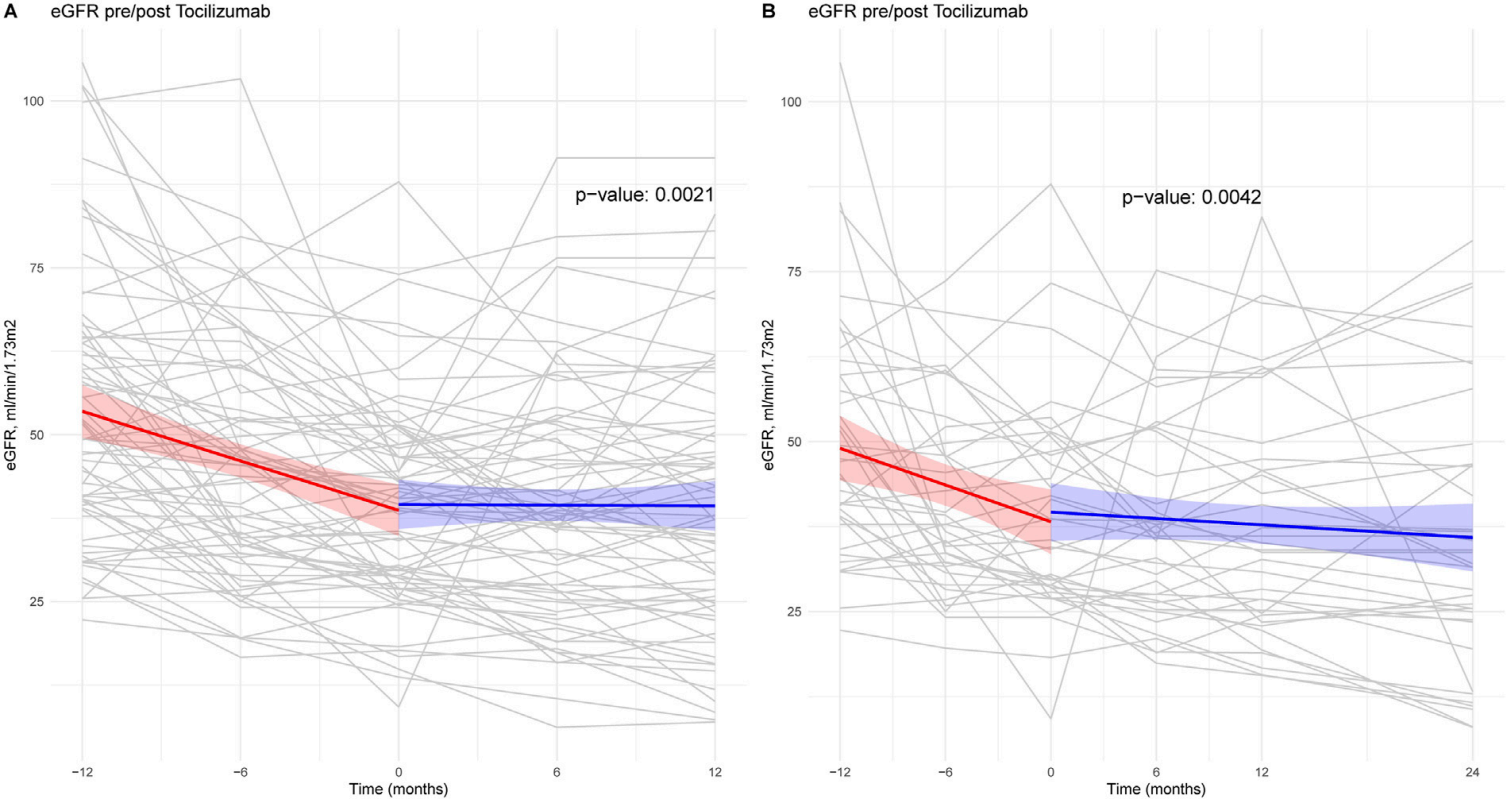
Estimated glomerular filtration rate trajectories before versus after Tocilizumab treatment. Panel **(A)** shows the mixed linear regression between – 12 months and +12 months post-tocilizumab. Panel **(B)** shows the mixed linear regression between – 12 months and +24 months post-tocilizumab. Grey curves represent patient’s eGFR evolution during each period of follow-up. Time “0” corresponds to the introduction of Tocilizumab to treat antibody-mediated rejection. The p-value for the comparison of the two models, indicating the statistical significance of the difference between the two periods: pre and post tocilizumab.

The linear mixed-effect model of the eGFR trajectory showed a significant difference between the pre- TCZ and post- TCZ period: -1.2 ± 0.2 vs. +0.03 ± 0.2 mL/min/1.73 m^2^/month, respectively; p = 0.001 (Figure 1A). When considering only the patients with 2 years of follow-up, eGFR trajectory decreased from −1.2 ± 0.2 mL/min/1.73 m^2^/month before TCZ versus −0.15 ± 0.1 mL/min/1.73 m^2^/months after 2 years TCZ, p < 0.001 (Figure 1B).

eGFR trajectory was then evaluated according to the Banff 2022 classification. In caAMR phenotype patients, the eGFR trajectory significantly decreased from −0.9 ± 0.3 mL/min/1.73 m^2^/month before TCZ to −0.2 ± 0.3 mL/min/1.73 m^2^/month after TCZ, p = 0.038), as shown in Supplementary Figure S1A. In MVI + DSA-C4d- patients, there was also a significant improvement of eGFR trajectory after TCZ introduction (−1.9 ± 0.5 mL/min/1.73 m^2^/month before TCZ versus +0.5 ± 0.5 mL/min/1.73 m^2^/month after TCZ, p = 0.007) (Supplementary Figure S1B).

In both centers, the improvement of eGFR trajectory post TCZ was significant: -1.4 ± 0.5 mL/min/1.73 m^2^/month before TCZ versus +0.1 ± 0.2 mL/min/1.73 m^2^/month after TCZ, p = 0.004 in Grenoble and 0.6 ± 0.3 mL/min/1.73 m^2^/month before TCZ versus −0.5 ± 0.2 mL/min/1.73 m^2^/month after TCZ, p = 0.03 in Bologna (Supplementary Figure S2).

**FIGURE 2:**
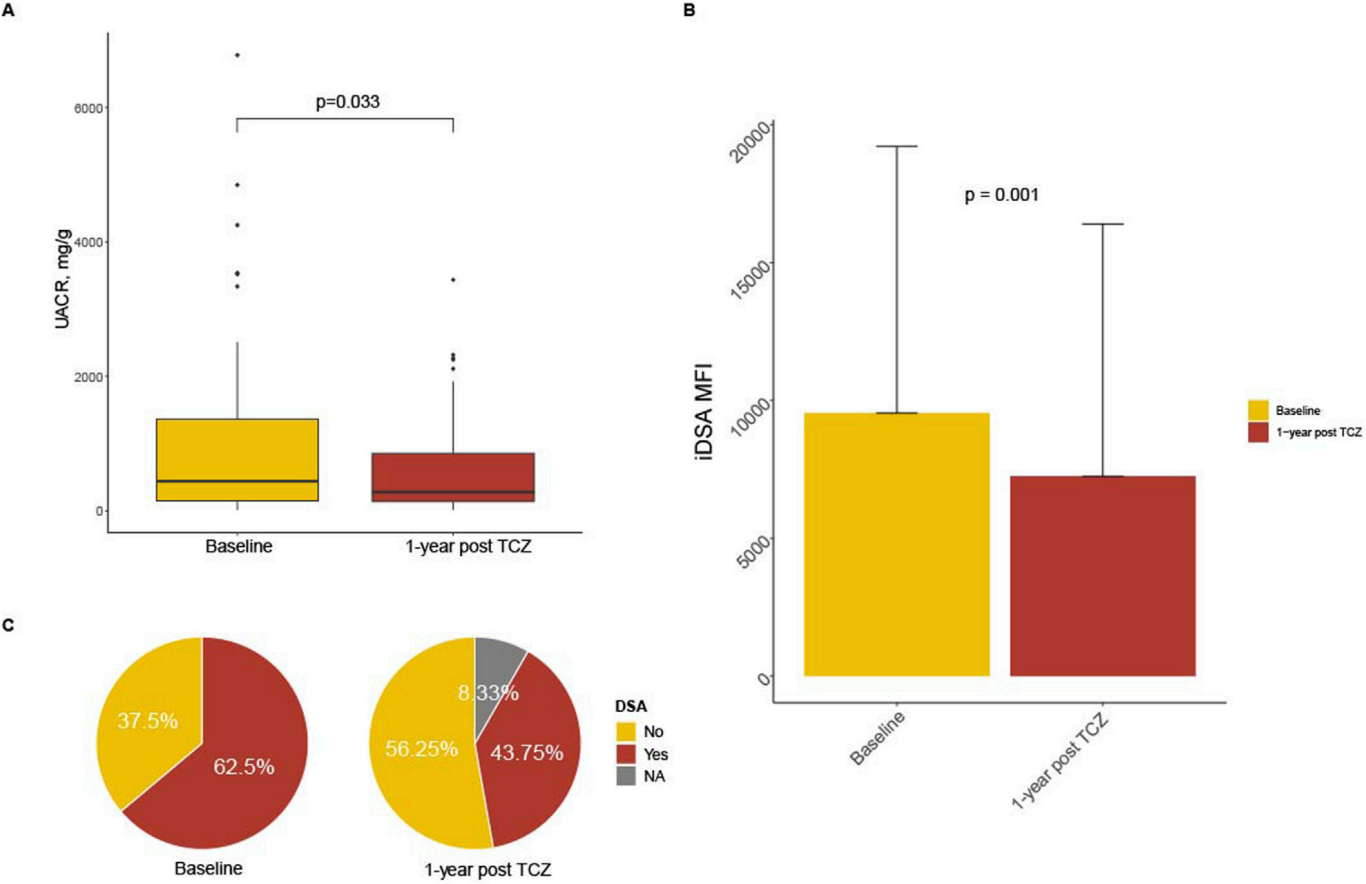
Evolution of Donor-specific antibodies (DSA) and albuminuria post tocilizumab (TCZ). Panel **(A)** shows boxplots of urinary albuminuria over creatininuria (mg/g) at baseline and at 1-year post TCZ. Panel **(B)** shows the MFI of the immunodominant DSA (iDSA) at the time of diagnosis (baseline) and after 1-year post TCZ (median and SD). Panel **(C)** chows Pie chart of the presence of at least one DSA at the time of diagnosis (baseline) and after 1-year post TCZ treatment.

In the whole cohort, albuminuria/creatininuria ratio declined from 1.0 ± 1.3 g/g to 0.6 ± 0.7 at 1-year after TCZ (p = 0.033; Figure 2A).

### Prediction of Response to Treatment

To evaluate the predictors of TCZ response, patients whose eGFR did not decrease (trajectory ≥0) between baseline and 1-year post treatment were defined as “responders” to TCZ, whereas patients whose eGFR decreased after 1-year were defined as “non-responders.” According to this definition, twenty-five (37.8%) patients were classified as “responders” and 41 (62%) patients were “non-responders”. In univariate analysis the factors that resulted to be significantly associated with TCZ response were younger age (OR = 0.97 [0.96–0.98], p = 0.035), lower chronic glomerulopathy lesions in the initial biopsy (OR = 3.45 [1.14–11.9], p = 0.035), and the MVI + DSA-C4d- phenotype (OR = 3.58 [1.24–10.8], p = 0.019). In the multivariate analyses, younger age (OR = 0.9 [0.91–0.99], p = 0.022), lower score of chronic glomerulopathy (OR = 4.49 [1.29–18.9], p = 0.025) and MVI + DSA-C4d- phenotype (OR = 5.25 [1.55–20.71], p = 0.01) remained significantly associated with the 1-year response to TCZ (Table 2).

**TABLE 2:** Univariate logistic regression and multivariate Cox regression analysis of factors associated with TCZ response.

Variable	Univariate analysis		Multivariate analysis	
	HR (95% CI)	*p*-value	HR (95% CI)	*p-*value
Age	0.97 [0.96–0.98]	<0.035	0.95 [0.91–0.99]	0.022
Gender (reference: female)	1.13 [0.78–1.64]	0.507		
Pre-emptive transplant	0.27 [0.03–1.49]	0.148		
Donor status	2.27 [0.61–23.10]	0.160		
Induction therapy (reference: thymoglobulin)	3.26 [0.72–8.84]	0.219		
Transplant glomerulopathy score	3.45 [1.14–11.9]	0.035	4.49 [1.29–18.96]	0.025
C4D deposition (yes/no)	0.60 [0.17–1.90]	0.406		
MVI + DSA-C4d- phenotype (reference: AMR phenotype)	3.58 [1.24–10.84]	0.019	5.25 [1.55–20.71]	0.010
iDSA MFI	1.0 [0.99–1.00]	0.906		
IL6 dosage	0.97 [0.84–1.09]	0.706		
eGFR	0.98 [0.95–1.01]	0.371		
Albuminuria	1.00 [0.99–1.00]	0.353		

Responder to TCZ are patients whose eGFR did not decrease (trajectory ≥0) between baseline and 1-year post treatment. TCZ: tocilizumab; *p < 0.05. Mean ± SD.

### Graft and Patient Survival

At 1-year post-TCZ, patient survival was 98.4% and graft survival was 93.75%. During the follow up, one patient died (due to gastric cancer 48 months after TCZ start and one patient discounted TCZ, documented by a 4-fold increase over the baseline of AST and ALT blood levels. At last-follow-up visit post-TCZ, patient survival was 98.4% and graft survival was 85.9%.

The patients who lost their graft during the first year were significantly older compared to the rest of the cohort (61.4 ± 15 vs. 47.9 ± 14 years-old, p = 0.016), were diagnosed for MVI after a longer period post-transplantation (16.4 years [12–20] versus 4.8 [1–10.3] years, p = 0.003) and had a higher UACR at the time of biopsy (1.5 ± 0.9 vs. 0.9 ± 1.3 g/g, p = 0.024). There was no difference in the initial histological severity between patients who developed graft loss and the others. All patients who experienced transplant failure had received their kidney grafts from deceased donors, while 9.4% of grafts of the rest of the cohort were from living donors, a difference that did not reach statistical significance (p = 0.180). At last follow-up visit of 19.7 [13–32] months, 15 patients (20.8%) had lost their graft after a mean time of 13.4 ± 10 months.

### DSA Changes After Starting Tocilizumab Therapy

At the beginning of TCZ treatment, the median MFI of iDSA was 9,537 [1,426–15,075] and, at 1-year post treatment, decreased to 7,250 [3,100–14,975] (p = 0.001) (Figure 2B). Forty (62.5%) patients had at least a positive circulating DSA before TCZ treatment and, at 1-year post treatment, 28 (38.9%) of patients had a positive DSA (p < 0.001) (Figure 2C). Most of those DSA were class II (84.3%). Patients referred as “responders” were more often DSA negative (72.5% versus 45.8%, p = 0.03), but the average MFI of the iDSA was not statistically different between “responders” and “non-responders”: 3,400 [1,265–19,175] versus 10,000 [2,912–13,900] respectively, p = 0.648. There was no statistical difference for DSA presence or iDSA MFI between patients who lost their graft versus those with a functioning graft at 2-year post- TCZ.

### Interleukin-6

Serum specimens were available for 25 patients before and at 1-year post-treatment. In these patients, IL-6 levels significantly increased at 1 year after treatment initiation (Supplementary Figure S3). Baseline IL-6 levels were tendentially higher (although not significant) the “non-responders” than in the “responders” (7.1 ± 10 versus 5.9 ± 3.1 pg/mL, p = 0.118). Lastly, there was no association between serum IL-6 levels at baseline and graft loss (not shown).

### Histological Evolution

**FIGURE 3:**
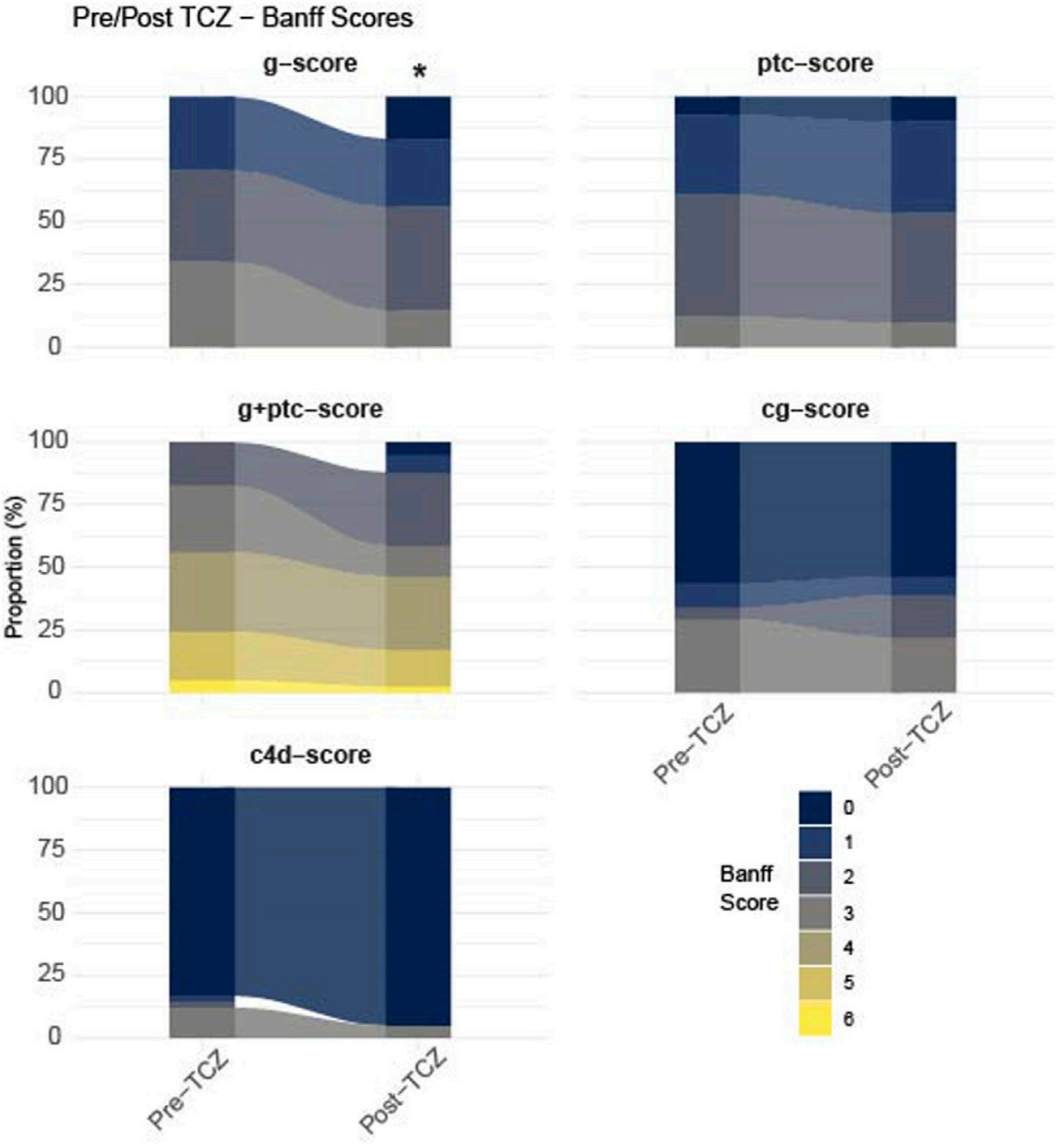
Plot of percentage of Banff Scores in biopsies before Tocilizumab (pre-TCZ) and 1-year after TCZ treatment (post-TCZ). Banff scores are g: glomerulitis, Ptc: Peritubular capilaritis, g + ptc: addition of g and ptc scores, c4d: complement deposition, cg: chronic glomerulopathy. *p-value = 0.014.

All patients of Grenoble had follow-up kidney biopsies. Figure 3 shows the evolution of g scores, ptc scores, g + ptc scores, c4d staining and chronic glomerulopathy lesions (cg) between the first biopsy with microvascular inflammation (MVI) diagnosis and 1-year post TCZ treatment. After 1-year in the TCZ group, a significant decrease was observed in the g score (p = 0.014) but not in the ptc, g + ptc, c4d and cg scores. Within the g-score, 29.3% had a score of 3 before TCZ vs. 22% at 1-year post-TCZ, p = 0.032.

### Tocilizumab Route of Administration

Within the cohort, 17 patients (26.6%) received TCZ subcutaneously during the first year of treatment. The evolution of eGFR post TCZ was followed in patients converted to sub-cutaneous TCZ within the first year and those who remained on intravenous infusion. In patients converted to sub-cutaneous injections, eGFR trajectory during the first year was – 0.21 ± 0.46 mL/min/1.73 m^2^/month, not statistically different from patients that received only intravenous TCZ: +0.15 ± 0.29 mL/min/1.73 m^2^/month, p = 0.521 (Supplementary Figure S4). Sub-cutaneous administration was not associated with a reduced response to TCZ (20.8% in the group of responder vs. 30% in the non-responder patients, p = 0.421).

### Tocilizumab Safety

Thirteen patients (20.3%) discontinued TCZ either because of histological or clinical stabilization (n = 7, 10.9%) or because of side effects (n = 6, 9.3%) at the end of follow-up (2 discontinued during the first year). The side effects that resulted in TCZ discontinuation were infections in five patients (peritonitis, cryptococcosis disease, CMV disease, tuberculosis and *campylobacter* infection), hepatotoxicity (1) within the first year and a DRESS (Drug reaction with eosinophilia and systemic symptoms syndrome) within the second year post-TCZ.

## Discussion

Few therapeutic options have proven their efficacy in caAMR and MVI + DSA-C4d- patients [30–32], that still remains among the main causes of long term graft loss. Others have found an increase of IL-6 mRNA transcripts in kidney allografts with rejection but not in healthy allografts [19]. Additionally, the addition of an IL6 blocker in association with co-stimulation blocker improved graft survival and decreased the rate of rejection in a cardiac transplantation mouse model [33]. Therefore, there is an emerging interest on targeting the IL-6/IL-6R pathway, which plays several roles in allograft inflammation [17].

TCZ, an IL-6 receptor blocker, has proven efficacy in the chronic/maintenance treatment of autoimmune diseases, like rheumatoid arthritis [34, 35]. Choi *et al.* [20] were the first to study the use of TCZ as a rescue therapy for caAMR in 36 KT recipients resistant to the standard of care. Graft- and patient-survival rates were excellent, respectively 80% and 91% at 6 years after AMR diagnosis. They also reported a significant decrease in DSA, C4d deposition and microvascular infiltration after TCZ treatment whereas the cg score and renal function remained stable. The first randomized controlled study with a direct IL-6 inhibitor (Clazakizumab) versus placebo in caAMR was published by Doberer *et al* [27]. Even though Clazakizumab arm showed no improvement of molecular and histological features of AMR over placebo, Clazakizumab patients had a significant decrease of DSA MFIs, MVI score and a slower decline of eGFR trajectories. This is consistent with our findings showing a significant decrease of eGFR loss after TCZ initiation.

When analyzing the factors associated with TCZ response, we showed that the response to TCZ was higher in those with less cg lesions, suggesting that TCZ may be more effective if initiated early in the setting of caAMR and MVI.

Our data highlighted a more marked effect when TCZ is used in the presence of fewer chronic lesions, in agreement with a previous randomized study on 30 patients with subclinical inflammation by Chandran *et al.* [36] who described histological improvement and increased regulatory T cells post-TCZ. Taken together, these data suggest that TCZ may be considered in the early stages of graft inflammation.

Our study revealed a global stabilization of eGFR already 1 year after TCZ initiation, which was statistically confirmed at 2 years post TCZ initiation. Moreover, the positive effect of TCZ was also corroborated by proteinuria reduction and decrease of g-score at 1 year.

The two cohorts presented some differences at baseline. In the Bologna’s cohort recipients were older, with longer transplant vintage at TCZ start worse chronic lesions, and the more frequent presence of DSA at the time of MVI diagnosis. Despite these differences, TCZ demonstrated the same efficacy in the two populations.

The presence of DSAs is strongly associated with kidney graft failure [37]. Here, we show a significant decrease of DSA number and of iDSA MFI at 1-year post treatment, consistently with the literature evidence of the role of IL-6 in promoting the production of HLA-DSA [38]. It can be postulated that treatment with TCZ, by lowering the titer of these antibodies, may improve graft survival. Our study is in line with other cohorts, where an overall decrease of iDSA MFI was noticed [39].

Elevated circulating IL-6 is observed in patients with inflammatory diseases [35, 40, 41] and the IL-6 levels correlate with disease activity. A pharmacological study [34] suggested that, under TCZ, serum IL6 trough level rises in rheumatic arthritis patients (1.55-fold) and in Castleman disease (23-fold). Likewise, at 1-year post treatment, we notice a 10-fold increase, which is strong evidence in favor of efficient IL-6 blockage by TCZ in KT recipients. It has been shown that the increase of IL-6 following TCZ is not attributed to increase secretion but to the inhibition of IL-6 receptor-mediated clearance by TCZ [34]. After blockade of the receptor by TCZ, the serum IL-6 level after TCZ may reflect the level of endogenous production and therefore the baseline level of inflammation. In our TCZ cohort, a higher level of serum IL-6 was not associated with clinical outcomes.

Our study is in line with other studies concerning TCZ in caAMR patients with an overall good tolerance, as only 2 patient discontinued the TCZ treatment within the first year and 4 patients at the end of follow-up because of possible related side effects [42, 43].

It is known that rejection is primarily caused by non-adherence to therapy; therefore, a treatment that adds to the already high number of pills taken and requires in-hospital administration could increase non-compliance. Although the trend in patients that received sub-cutaneous TCZ was a lower eGFR at 1-year post treatment compared to intravenous patients, we did not find a significant difference in the eGFR trajectory. This aspect could be relevant in improving compliance and it could benefit patients with poor venous access. Long follow-up data are needed to confirm the equally effectiveness between sub-cutaneous and intravenous administration, as it was shown in rheumatoid arthritis patients [44].

Our study has some limitations to be acknowledged, including its retrospective nature. Moreover, patient’s criteria of inclusion (i.e., histological diagnosis of MVI) may be heterogeneous as it includes caAMR and MVI + DSA-C4d- phenotypes associated with different renal prognosis. However, our inclusion criteria based on MVI, allowed to show that the response to TCZ was significantly associated in multivariate analysis with MVI + DSA-C4d- phenotype. These results highlight the possible efficacy of anti-IL6 therapies beyond humoral-mediated injuries but may be rejection mediated by immune cells such as NK cells or T-cells [11, 13]. TCZ have been shown to reduce NK cells and promote regulatory T [45], and to enhance NK cells cytotoxicity and cytokine production [46]. Moreover, IL-6 is involved in T cells mediated endothelial injury [47]. We may hypothesis that, by blocking IL-6, TCZ reduce NK mediated inflammation and endothelial injury in the graft, promote graft tolerance and reduce endothelium activation. The inflammation in non-humoral rejection may be driven by IL-6, which TCZ directly targets. Another limitation of this study is the absence of a matched control cohort, which restricts the strength of causal interpretations. Although we explored retrospective matching strategies using key clinical variables such as baseline eGFR, rejection severity, DSA status, and age, the small sample size and heterogeneity of our cohort—including both caAMR and MVI cases with or without DSA—made this approach unfeasible without introducing significant bias. Future prospective studies with matched control groups will be essential to confirm and extend these findings.

In a nutshell, this study suggested that first-line therapy with TCZ for patients with MVI histological feature is associated with an improvement of eGFR trajectories, and reduced DSA number and MFI. Response to TCZ was higher in younger patient, within the MVI + DSA-C4d- group and when associated with less chronic glomerulopathy lesions, suggesting that TCZ may be more effective as started early during MVI evolution, and may maintain longer the benefits for renal function. Initial IL-6 levels do not seem associated with clinical outcomes. Despite the failure of the IMAGINE trial using Clazakizumab, large randomized controlled trials on IL-6 receptor blockade such as the INTERCEPT study are needed to fully assess the efficacy of this strategy.

## Data Availability

The raw data supporting the conclusions of this article will be made available by the authors, without undue reservation.

## Abbreviations

AMR, Antibody-Mediated Rejection; caAMR, chronic active AMR; cg, Chronic Glomerulopathy; CKD-Epi, Chronic Kidney Disease-Epidemiology collaboration equation; CLZ, Clazakizumab; CMV, Cytomegalovirus; DSA, Donor-Specific Antibody; eGFR, estimated Glomerular Filtration Rate; g, glomerulitis; HLA, Human Leukocyte Antigen; iDSA, immunodominant Donor-Specific Antibody; IL-6, Interleukin-6; IL-6R, Interleukin-6 Receptor; KT, Kidney Transplantation; MFI, Mean fluorescence Intensity; Ptc, Peritubular capillaritis; RCT, Randomize Clinical Trial; TCZ, Tocilizumab; UACR, Urine Albumin‐To‐Creatinine Ratio.
