# Supplementary material for: Tocilizumab-Based Treatment of Microvascular Inflammation in Kidney Transplant Recipients: A Retrospective Study
Source: Transpl Int. 2025 May 16;38:14502. doi: 10.3389/ti.2025.14502 (PMC12124137; doi:10.3389/ti.2025.14502)
Supplement: Supplementary file 1 [file DataSheet1.PDF]

Tocilizumab in Kidney transplant recipients with chronic active antibody-mediated rejection or microvascular inflammation

1. **Supplementary data**

Supplemental Table 1: Baseline characteristics of patients according to center.

	Patient with TCZ as a first line therapy for caAMR with at least 1 year of follow-up N = 64		
	Grenoble N= 39	Bologna N= 25	p-value
Age – years	44.8 ± 15	53.3 ± 11	0.02
Female Gender - N (%)	16 (41.0%)	10 (40%)	0.93
Pre-emptive transplantation – N(%)	5 (12.8%)	1 (4.0%)	0.24
Nephropathy– N(%)<br>PKD<br>Diabetes<br>Vascular disease<br>Autoimmune<br>Unknown<br>Other	 8 (20.5%)<br>4 (10.3%)<br>4 (10.3%)<br>5 (12.8%)<br>13 (33.3%)<br>5 (12.8%)	 7 (28.0%)<br>2 (8.0%)<br>0 (0%)<br>2 (8.0%)<br>9 (36.0%)<br>5 (20%)	0.58
Induction therapy<br>antithymoglobulin<br>Basiliximab	 39 (100%)<br>0	 8 (44.4%)<br>12 (66.7%)	<0.001
Living donor – N (%)	8 (20.5%)	3 (12.0%)	0.38
DSA at the time of biopsy – N (%)	19 (48.7%)	21 (84.0%)	0.01
Serum creatinine at the time of biopsy - µmol/L	177 ± 99	185 ± 52	0.44
eGFR at the time of biopsy – mL/min/1.73m^2	40 ± 15	37 ± 16	0.13

Albuminuria at the time of biopsy – g/g of creatininuria	1.0 ± 1.3	0.9 ± 1.3	0.61
Time after transplant - months	35 [12 – 66]	154 [61 – 221]	<0.001
Immunosuppression at the time of biopsy			
- Tacrolimus	39 (100%)	11 (45.8%)	0.07
- Cyclosporine	0 (0%)	12 (50%)	0.02
- MMF	35 (89.7%)	17 (73.9%)	0.84
- Everolimus	4 (10.3%)	3 (13.0%)	0.05
Histology			
- Glomerulitis score ≥ 2	28 (71.8%)	3 (12%)	<0.001
- Peritubular capillaritis ≥ 2	15 (38.5%)	7 (28.0%)	0.39
- IFTA	0	3 (12.0%)	NS
- Transplant glomerulopathy	20 (51.3%)	14 (56.0%)	0.71
- C4d positivity	7 (17.9%)	10 (40.0%)	0.42
Banff 2022 classification			
- MVI DSA-C4d-, cg >0	15 (38.5%)	4 (16.0%)	0.02
- caAMR	24 (61.5%)	21 (84.0%)	0.11

AMR: anitbody mediated rejection; DSA: Donor-specific antibody; cg: chronic glomerulopathy; eGFR: estimated glomerular filtration rate; IFTA : interstitial fibrosis and tubular atrophy; MMF : mycophenolate mofetil/mycophenolic acid; MVI: microvascular inflammation (g+ptc ≥2); PKD: polycystic kidney disease; TCZ: tocilizumab. Mean ± SD

2. Supplementary Figures

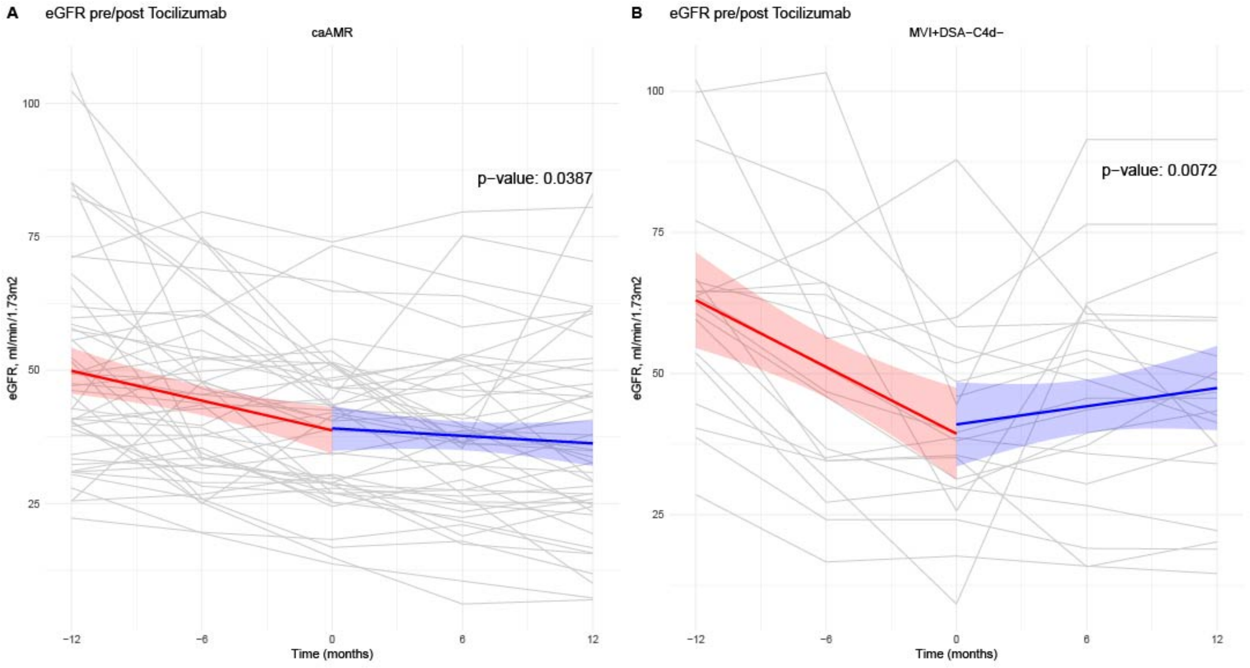

Supplementary Figure 1: Estimated glomerular filtration rate slopes before versus after Tocilizumab treatment according to Banff 2022 patient phenotype. Panel A shows the mixed linear regression in baseline caAMR patients phenotype. Panel B shows the mixed linear regression in MVI+DSA-C4d- patient phenotype. Grey curves represent patient's eGFR evolution during each period of follow-up. Time "0" corresponds to the introduction of Tocilizumab to treat antibody-mediated rejection. The p-value for the comparison of the two models, indicating the statistical significance of the difference between the two periods: pre and post Tocilizumab.

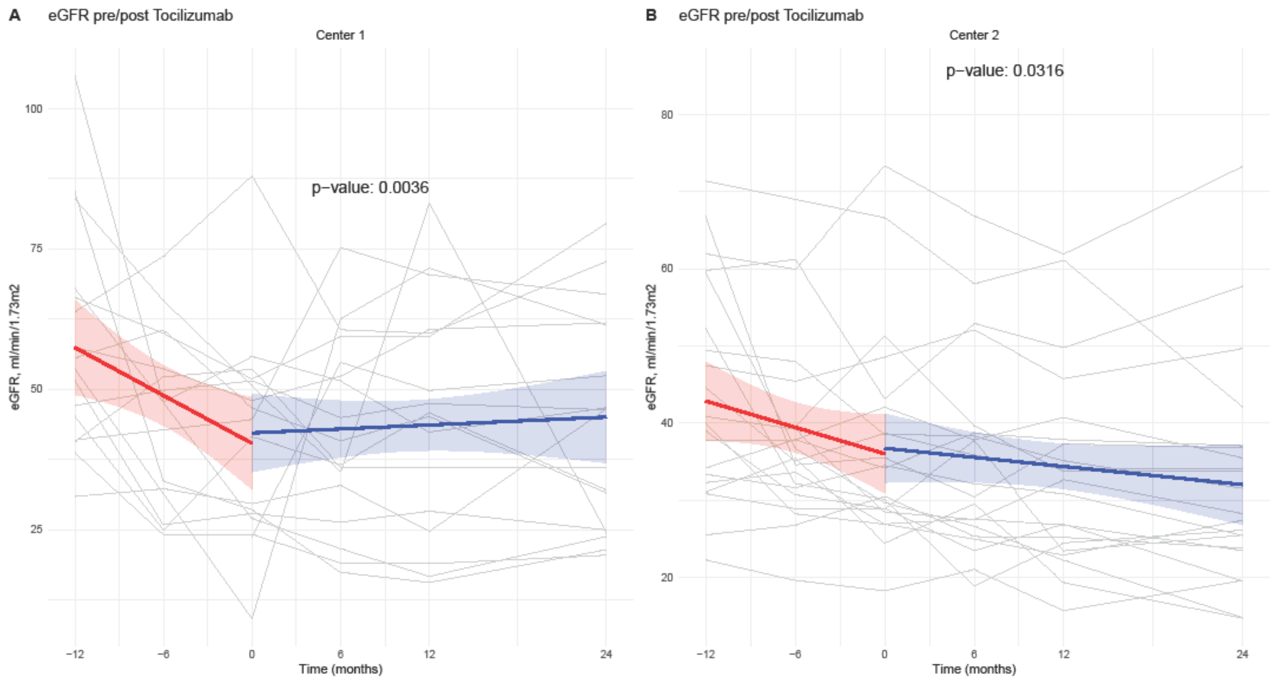

Supplementary Figure 2: Estimated glomerular filtration rate slopes before versus after Tocilizumab treatment according to the center. Grey curves represent patient's eGFR evolution during each period of follow-up. Time "0" corresponds to the introduction of Tocilizumab to treat antibody-mediated rejection. The lines represent the linear regression models fitted to the data points for each period. Center 1 is Grenoble; Center 2 is Bologna. The p-value for the comparison of the two models, indicating the statistical significance of the difference between the two periods: pre and post Tocilizumab.

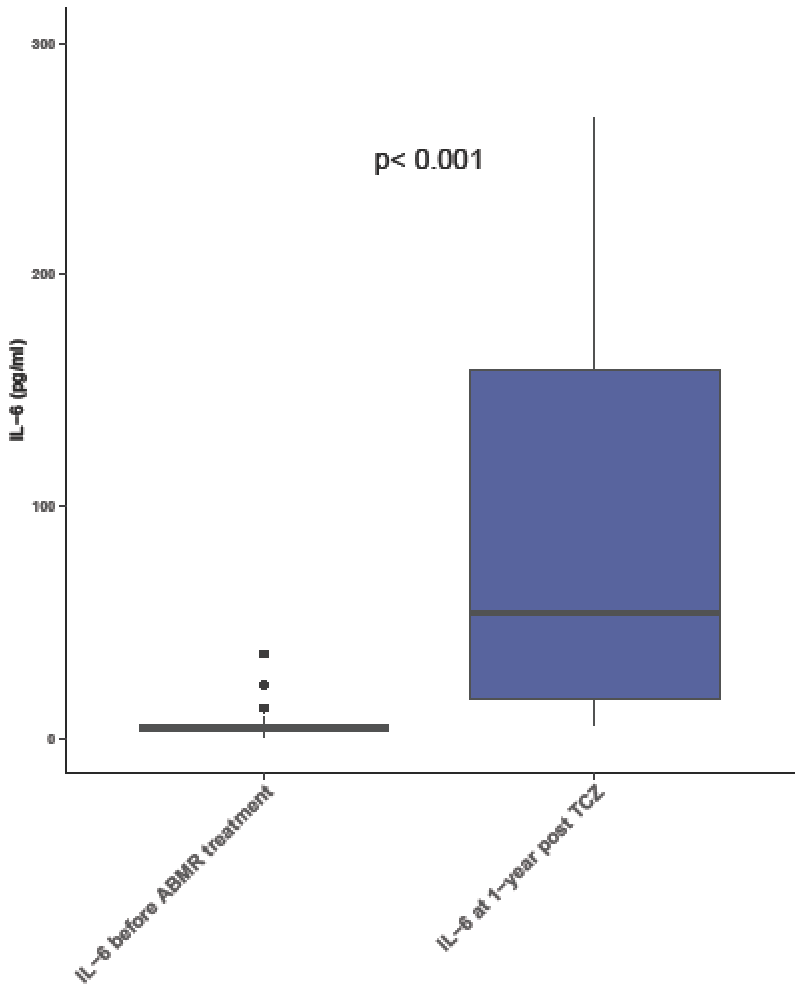

Supplementary Figure 3: Boxplots of Interleukin-6 (IL6) (pg/ml) in the tocilizumab-treated. IL-6 dosages were performed in available samples at baseline and at 1-year post treatment.

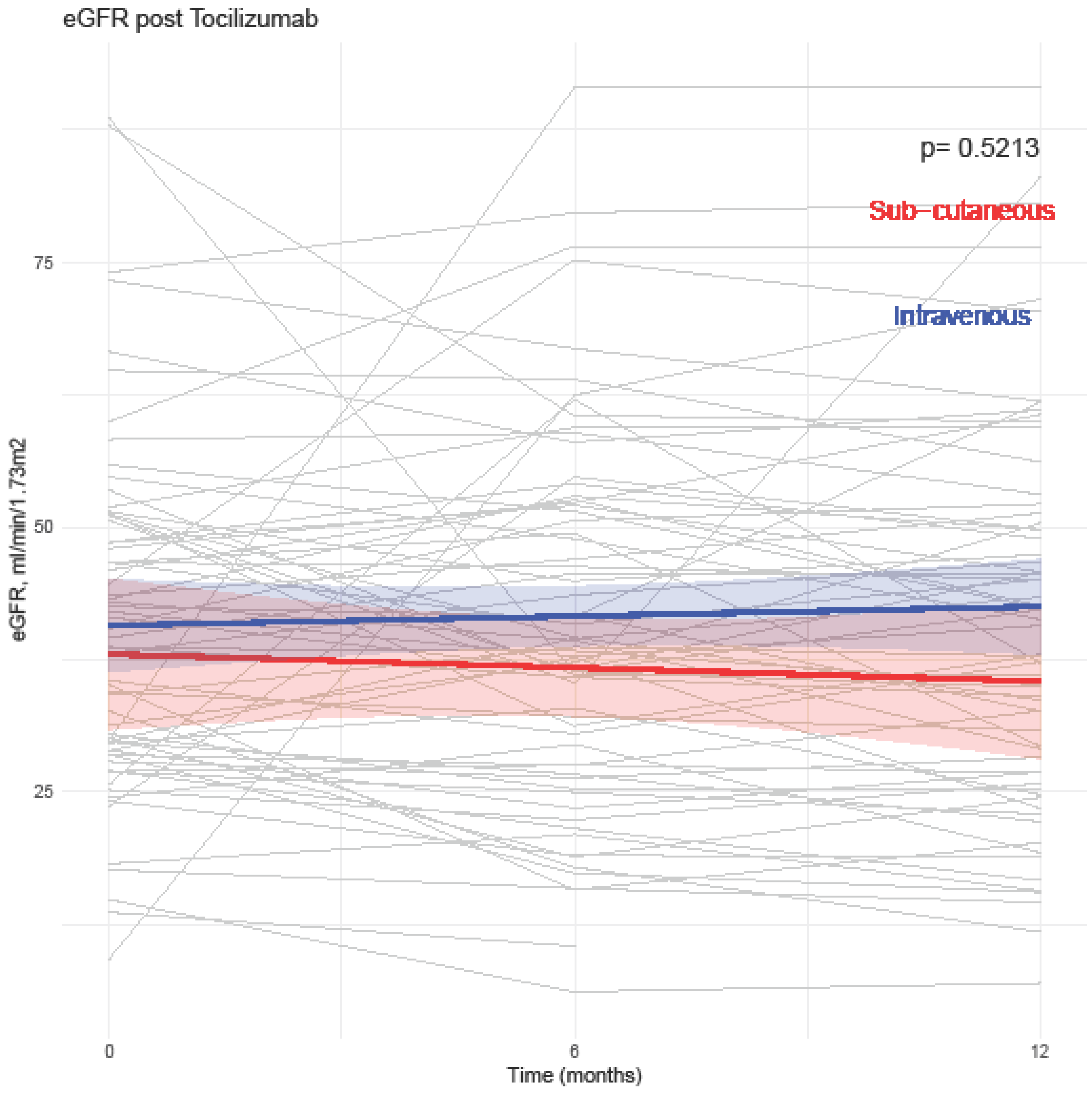

Supplementary Figure 4: Estimated glomerular filtration rate slopes after Tocilizumab treatment between sub-cutaneous and intravenous tocilizumab administration. Grey curves represent patient's eGFR evolution during each period of follow-up. Time "0" corresponds to the introduction of Tocilizumab to treat antibody-mediated rejection. The p-value for the comparison of the two models, indicating the statistical significance of the difference between the two groups: sub-cutaneous versus intravenous only during the first year of treatment.
